# Gut microbiome profiles of two limestone forest-dwelling langurs show stronger influence of host-associated factors than environment

**DOI:** 10.3389/fmicb.2026.1953526

**Published:** 2026-09-30

**Authors:** Wen Xie, Shiyi Lu, Zheng Liu, Jingjin Zheng, Fengxiang Mo, Kechu Zhang, Zhonghao Huang

**Affiliations:** 1 Key Laboratory of Ecology of Rare and Endangered Species and Environmental Protection (Guangxi Normal University), Ministry of Education, Guilin, China; 2 The Chongzuo White-Headed Langur Field Observation and Research Station of Guangxi, Chongzuo, China; 3 Guangxi Forest Resources and Environment Monitoring Center, Nanning, China; 4 Key Laboratory of Mountain Biodiversity Conservation, Education Department of Guangxi Zhuang Autonomous Region, Yulin Normal University, Yulin, China

**Keywords:** gut microbiome, host-associated factors, langurs, limestone forests, metabolism

## Abstract

**Background:**

Host-associated factor and the environment play crucial roles in shaping the composition and function of the gut microbiome. Considerable debate exists regarding the relative influence of host-associated and environmental factors on the gut microbiome. To determine the relative importance of host-associated factors and environmental factors in langurs, we employed metagenomic sequencing to examine differences in the gut microbiome of the white-headed langurs (*Trachypithecus leucocephalus*) and François’ langurs (*T. francoisi*).

**Results:**

The results revealed that the alpha diversity of the gut microbiome significantly differed between white-headed langurs and François’ langurs in similar habitats. However, there was no significant variation in the alpha diversity between the wild and captive François’ langurs. Similarly, the proportion of Bacillota differed between the langur species, with a higher proportion in the white-headed langurs than the François’ langurs. In addition, significant separation in gut microbiome functions also occurred between the two species. The functional genes related to metabolism (e.g., carbohydrate metabolism and amino acid metabolism) and Glycoside Hydrolases in wild François’ langurs were more abundant than in the other langurs. The results of PCoA showed that the pathways were significantly separated between white-headed langurs and François’ langurs at four levels in the KEGG database, whereas the pathways showed no significant differences between the wild and captive François’ langurs except for module level. The proportion of ARGs related to multidrug resistance was higher in wild François’ langurs, likely associated with the utilization of poultry manure and antibacterial biocides in the langur’s habitat.

**Conclusion:**

Our study demonstrates that the host-associated factors have stronger influence on shaping the gut microbiome of langurs than environmental factors. These findings provide data on the relative contributions of host-associated factors and environmental factors to the gut microbiome of langurs and identify potential driving factors for antibiotic resistance genes. In addition, these findings provide crucial insights into the influence of host-associated factors on the gut microbiome, offering a theoretical basis for future langur conservation strategies.

## 1 Introduction

The gut microbiome plays an essential role in host health and adaptability, supporting digestion, nutrient absorption, immune modulation, and metabolism (Zhu et al., 2010). Diversity, stability, flexibility, and resilience are regarded as primary characteristics of the gut microbiome, reflecting its capacity to respond to environmental changes (Lozupone et al., 2012; Liu et al., 2023). The dominant gut microbiota in non-human primates belong to Bacillota (formerly Firmicutes), which are important in the digestion of dietary fiber (Liu et al., 2023; Mo et al., 2023; Sun et al., 2023a). Specifically, folivorous primates depend heavily on their gut microbiome to break down dietary fiber into short-chain fatty acids (SCFAs) (Shabat et al., 2016). The gut microbiomes of primates are shaped by a range of factors, including host genetics, dietary composition, and habitat conditions (Barelli et al., 2015; Amato et al., 2019; Chen et al., 2023; Sun et al., 2023a; Liu et al., 2024). For example, the composition and function of the gut microbiome are substantially shaped by host genetics (Goodrich et al., 2014; Bubier et al., 2021; Yang et al., 2021). Previous studies have demonstrated that the initial colonization of the gut microbiome is determined by host genetics, with each individual harboring host-specific microbial lineages (Dąbrowska and Witkiewicz, 2016). However, environmental factors also play an essential role in shaping the composition and function of the gut microbiome. For example, intersite differences in the gut microbiome are associated with habitat quality in François’ langurs (*Trachypithecus francoisi*), with individuals in lower-quality habitats exhibiting a higher relative abundance of Bacillota and more enriched functional genes related to amino acid metabolism and metabolic pathways than those in higher-quality habitats (Qiu et al., 2026). These fluctuations in the gut microbiome even occur at a short temporal scale, as exemplified by the seasonal dynamics of primate gut microbiota in response to plant phenology, likely reflecting seasonal shifts in diets and food availability (Chen et al., 2023).

Views on the relative importance of host-associated and environmental factors in shaping the gut microbiome of animals remain inconsistent in current reports (Carmody et al., 2015; Rothschild et al., 2018; Amato et al., 2019; Knowles et al., 2019). Previous studies have demonstrated that host-associated factors outweigh environmental factors in shaping the gut microbiome, and that the initial composition of the gut microbiome is shaped by host genetics rather than being acquired from the environment (Dąbrowska and Witkiewicz, 2016; Amato et al., 2019; Yang et al., 2021). For instance, sympatric François’ langurs and rhesus macaques (*Macaca mulatta*) exhibited marked differences in gut microbial composition, despite both species consuming young leaves and sharing similar dietary diversity (Huang et al., 2010; Tang et al., 2016; Chen et al., 2020). This difference might be related to the impact of host genetics, as these species exhibit distinct genetic backgrounds and considerable phylogenetic distances (Chen et al., 2020). A study of small mammal species of three common genera, including mice (*Apodemus* spp.), voles (*Microtus* spp.), and shrews (*Sorex* spp.), showed that the gut microbiota of individuals differed among closely related species at the same site, whereas individuals of the same species from different locations had similar microbiota (Knowles et al., 2019). Although shared habitat patterns lead to convergence in microbial composition among closely related species, the host plays a dominant role in determining the composition of the gut microbiota (Knowles et al., 2019). Conversely, some studies indicate that environmental factors have a stronger effect on the gut microbiome of animals than host-associated factors (Rothschild et al., 2018; Lee et al., 2023). In fact, the gut microbiome of animals is shaped by diet, which is widely regarded as a key factor in structuring its composition and function (Carmody et al., 2015; Rothschild et al., 2018). For example, the gut microbiome plasticity allows it to adapt to changes in the host diet under varying environmental conditions in golden snub-nosed monkeys (*Rhinopithecus roxellana*) (Zhang M. et al., 2025), as reported in the case of François’ langurs (Mo et al., 2023) and the musk deer (*Moschus berezovskii*) (Li et al., 2017). Animals with similar diets have more similar gut microbial communities, which is considered evidence of convergent evolution among animals (Ley et al., 2008; Hale et al., 2018). A study of allopatric mountain gorillas (*Gorilla beringei beringei*) and western lowland gorillas (*G. gorilla gorilla*) showed that several traits of gut microbiomes and metabolomes converge under the same dietary constraints (Gomez et al., 2016). Similarly, sympatric Tibetan antelope (*Pantholops hodgsonii*) and blue sheep (*Pseudois nayaur*) show a similar diversity and core microbiota (Liu et al., 2025). According to phylogenetic analysis, gut microbiota may undergo convergent evolution due to dietary variation (Liu et al., 2025). At the temporal scale, the gut microbiome differs seasonally in composition and diversity, likely resulting from dietary shifts in animals (Chen et al., 2023; Li Q. et al., 2023; Zhang M. et al., 2025).

The lifestyles of wild and captive animals have notable distinctions, representing a substantial change in environmental conditions (McKenzie et al., 2017). Captivity can have an adverse effect on the gut microbiome of animals, which may lead to imbalances in microbial composition and function (Petersen and Round, 2014; Clayton et al., 2016; Frankel et al., 2019). Specifically, the captive environment may have limitations in providing the specialized diets and essential nutrients required by primates, resulting in reduced microbial diversity and potentially limiting their ability to obtain sufficient nutrients in captivity (Wasimuddin et al., 2022). For instance, compared with wild monkeys, captive Guizhou snub-nosed monkeys (*R. brelichi*) exhibit decreased gut microbial diversity and reduced abundances of plant polysaccharide-degrading bacteria (Lachnospiraceae and Ruminococcaceae), directly correlating with simplified captive diets (Hale et al., 2019). However, some captive primates demonstrate enhanced microbial diversity when provided with enriched diets (Wang et al., 2021; Wang et al., 2023; Feng et al., 2024). For example, rhesus macaques in provisioning environments exhibit elevated microbial richness compared with wild populations, a pattern similar to that observed in Japanese macaques (*M. fuscata*) and Javan slow lorises (*Nycticebus javanicus*) (Cabana et al., 2019; Chen et al., 2019; Lee et al., 2019). Likewise, captive golden snub-nosed monkeys show not only increased alpha diversity and Prevotella/Bacteroides ratios but also alterations in metabolic pathways, including decreased pentose phosphate pathway activity and enhanced glutamate biosynthesis, which may be linked to the dietary composition of captive monkeys (Wang et al., 2021).

The François’ langurs and white-headed langurs (*T. leucocephalus*) are distributed in limestone forests in southwest Guangxi, China, and are listed as endangered and critically endangered species, respectively, on the IUCN Red List (Bleisch and Yongcheng, 2020; Nadler et al., 2020; Li B. G. et al., 2024). The limestone langurs include Delacour’s langur (*T. delacouri*), black langur (*T. ebenus*), Lao langur (*T. laotum*), François’ langur, Cat Ba langur (*T. poliocephalus*), Hatinh langur (*T. hatinhensis*), and white-headed langur (Groves, 2007; Zhou and Huang, 2021). The François’ langurs and white-headed langurs are representative species of the genus *Trachypithecus*, these species are two of seven limestone langurs restricted to habitats characterized by karst topography (Groves, 2007). They are closely related and exhibit great morphological similarity, making the white-headed langur once being regarded as a subspecies of the François’ langurs (Li and Ma, 1980). However, a previous study revealed that the white-headed langur diverged from the François’ langur 0.46–0.27 million years ago (Liu et al., 2013). These langurs are typically folivorous, preferring young leaves and fruits in the rainy seasons and relying heavily on mature leaves as fallback foods during dry months (Li et al., 2016; Chen et al., 2023).

In this study, we employed metagenomic sequencing to explore the composition and function of the gut microbiome in the white-headed langurs and François’ langurs, aiming to examine the influences of host-associated and environmental factors on shaping the gut microbiome. Due to the absence of host genetic variation or genotyped individuals, we focused on the effects of host-associated and environmental factors on the gut microbiome. Host-associated factors, including digestive tract morphology, host phylogeny, and ecological and functional traits, operate on evolutionary timescales and remain relatively stable (McCord et al., 2014; Amato et al., 2019; Knowles et al., 2019). By contrast, environmental factors, such as seasonal variation in dietary composition and habitat, act primarily on ecological timescales and exhibit high plasticity (Gomez et al., 2016; Wang et al., 2024). Exploring the influence of host-associated and environmental factors on the gut microbiome could enhance our understanding of the significance of primate gut microbiota in adapting to environmental changes, thereby providing actionable baselines for population conservation.

## 2 Methods

### 2.1 Study sites, fecal sample collection and ethics statement

Fecal samples were collected in the Guangxi Chongzuo White-Headed Langur National Nature Reserve (107°16′53″–107°59′46″E, 22°10′43″–22°36′55″N) and the Wuzhou Langur Breeding Center, Guangxi, China. The nature reserve is 400–600 m above sea level and is covered by karst forests, which is vulnerable to human disturbance (e.g., sugarcane plantations, roads, and settlements) (Guangxi Forestry Department, 1993). These limestone forests are characterized by typical seasonal forests and steep cliffs, leading to a shortage of surface water and poor vegetation in these areas (Huang and Li, 2005). The habitat of the wild François’ langurs has high diversity of woody plants and high plant density in the nature reserve (Qiu et al., 2026). The dominant plant species are Moraceae, Fabaceae, and Malvaceae at the family level, whereas the plants with the highest dominance include *Streblus tonkinensis*, *Vitex kwangsiensis*, and *Sinosideroxylon pedunculatum* (Qiu et al., 2026). The Wuzhou Langurs Breeding Center has more than 300 François’ langurs provisioned in captivity (Shi et al., 2014). Captive François’ langurs were separately provisioned in cages that were divided into an inner chamber (4 m × 2 m × 3 m) and an outer chamber (4 m × 4 m × 4 m). There were a window and a door connecting the inner and outer chambers. The captive langurs completely relied on provisioned foods, including cakes (24.73%), fruits (18.28%), kernels (3.23%), and leaves (53.76%) (Mo et al., 2023).

This study was conducted in compliance with the guidelines of the Institutional Animal Care and Use Committee (IACUC) and was approved by the Laboratory Animal Care and Animal Ethics Committee of Guangxi Normal University (permit no. 202103-032). The study was conducted with permission from the Administration Center of Guangxi Chongzuo White-headed Langur National Nature Reserve and the Wuzhou Langur Breeding Center, Guangxi, China. This study did not involve any animal tissues. All fecal samples were collected after the animals had left the area to avoid a stress reaction. The wild langurs typically use caves or cliffs as sleeping sites and defecate before departing in the morning (Huang and Li, 2005). Before collecting samples, we usually tracked langurs to determine their sleeping sites and then collected fresh fecal samples on the following morning. However, we were unable to conduct individual identification for the wild langurs. We collected samples from captive langurs when they defecated in the cage in the morning, with individual identification. During sample collection, we used sterile bamboo sticks to collect the middle portion of the fresh feces. We collected fecal samples that were separated by more than 2 m to avoid potential collecting feces from the specific individual. Each sample was 3–5 g and was immediately placed into sterile tubes, then stored in a dry icebox. All samples were transferred to an −80 °C ultra-low-temperature refrigerator for storage until laboratory analysis. In this study, we collected a total of 27 fecal samples, including 9 samples from wild white-headed langurs and 10 samples from wild François’ langurs in Chongzuo during November 2020, and 8 samples from captive François’ langurs in Wuzhou during December 2020 (Supplementary Table S1).

### 2.2 DNA extraction and metagenomic sequencing

We used the E.Z.N.A.^®^ Soil DNA Kit (Omega BioTek, Norcross, GA, United States) to extract bacterial DNA from all fecal samples. The quality of the extracted DNA was assessed using 1% agarose gel electrophoresis, and its concentration and purity were quantified using a NanoDrop 200 ultraviolet–visible light spectrophotometer (Thermo Fisher Scientific, Wilmington, DE, United States). The extracted DNA was fragmented to an average size of 400 bp using a Covaris M220 ultrasonic crusher (Gene Company Limited, China) for paired-end library construction. DNA fragments underwent end-repair, poly-A tailing, bead-based cleanup, and size-selection screening to obtain complete adapter-ligated products. The NEXTFLEX^™^ Rapid DNA-Seq Kit was applied to construct libraries, enrich templates, and conduct sequencing on the Illumina NovaSeq platform.

Fastp (https://github.com/OpenGene/fastp, v 0.23.0) was utilized to remove the 3′ and 5′ adapters and filter out poor-quality sequences, including reads with a length < 50 bp, an average quality score < 20, or those containing N bases, while retaining high-quality PE and single-end reads. Reads were aligned to the host DNA sequences by BWA (http://bio-bwa.sourceforge.net/, v 0.7.17) to exclud contaminated reads with high similarity. We utilized MEGAHIT (https://github.com/voutcn/megahit, v 1.2.9) to splice and assemble the quality-controlled short-fragment sequences, retaining assembly results with a length ≥300 bp. The open reading frames (ORFs) from each assembled contig were predicted by Prodigal (https://github.com/hyattpd/Prodigal, v 2.6.3), and genes with a length of < 100 bp were excluded. The remaining genes were then translated into amino acid sequences to obtain gene prediction results. Clustering was performed on the gene sequences predicted from the samples using CD-HIT (https://github.com/weizhongli/cdhit/, v 4.6.1) to construct a non-redundant gene set, ensuring that the sequence identity and cluster coverage were both > 0.9. To determine gene abundance in each sample, we compared high-quality reads with non-redundant genes via SOAPaligner (https://github.com/ShujiaHuang/SOAPaligner, v soap2.21release) with 95% sequence identity. The non-redundant gene sets were searched and compared using Non-Redundant Protein Sequence (NR, v 20241007) database, Kyoto Encyclopedia of Genes and Genomes (KEGG, v 20241007, http://www.genome.jp/kegg/) database, and the Comprehensive Antibiotic Resistance Database (CARD, v 3.2.9, https://card.mcmaster.ca) for annotation using Diamond software (v 2.0.13), and the Carbohydrate-Active enzymes (CAZymes, v 12, http://bcb.unl.edu/dbCAN2/download/Databases/) database was used for annotation with HMMER (http://hmmer.org/, v 3.1b2).

### 2.3 Statistical analysis

In this study, the Shannon and Simpson indices were used to reflect community diversity, and the Ace and Chao indices were used to reflect the community richness of the gut microbiome. The alpha indices were converted to the form of log_10_(X + 1) and visualized as box plots. The Principal Co-ordinates Analysis (PCoA) based on Bray-Curtis distance was performed to determine differences in the gut microbiome between the two groups, with Adonis test. In addition to PCoA, we constructed generalized linear mixed models (GLMMs) to examine the differences between the two groups in the composition and function of the gut microbiome. Specifically, the alpha diversity indices, the proportion of community composition at the phylum and family levels, and the relative abundance of taxa at different functional annotation levels were set as the response variables. Species (white-headed langurs and François’ langurs) or site (wild and captive François’ langurs) was set as the fixed factors, and the number of fecal samples collected in each group was set as a random factor in the model. In addition, we examined differences between models with and without the fixed factors, using ANOVA to detect the effects of the fixed factors on each dependent variable. All the statistical analyses was performed in R (v 4.5.1), with the significance level set at *p* < 0.05.

## 3 Results

### 3.1 Metagenomic sequencing results

Metagenomic sequencing generated 1,262,228,970 raw reads from 27 fecal samples. After quality control, we obtained 1,256,152,716 clean reads, with an average clean Q20 value of 99.30 ± 0.18% and an average clean Q30 value of 96.74 ± 0.12%. Comparison of the non-redundant gene set against the NR database revealed 166 phyla, 276 classes, 461 orders, 937 families, 3,555 genera, 16,249 species in the gut microbiome of the white-headed langurs and François’ langurs. The sequencing data statistics are shown in Supplementary Table S2.

### 3.2 Composition of the gut microbiota based on metagenomic sequencing

The gut microbiota of all samples at the phylum level was predominantly composed of Bacillota, and Bacteroidota (formerly Bacteroidetes), which accounted for more than 80% of the total gut microbiota. The predominant gut microbiota were Bacillota (85.83% ± 2.60%), Bacteroidota (3.35% ± 0.78%), and Mycoplasmatota (2.79% ± 0.45%) in the white-headed langurs (Figure 1A). The dominant taxa of the wild François’ langurs were Bacillota (75.27% ± 5.17%), Bacteroidota (15.73% ± 3.93%), and Verrucomicrobiota (3.90% ± 4.96%) (Figures 1A,B). Furthermore, the gut microbiota of captive François’ langurs was dominated by Bacillota (76.84% ± 2.47%), Bacteroidota (7.36% ± 3.30%), and Mycoplasmatota (6.33% ± 2.46%) (Figure 1B). Significant differences were observed in their gut microbiome (Figures 1C,D). Specifically, the relative abundance of Bacillota in the white-headed langurs was significantly higher than that in wild François’ langurs; however, no significant difference was observed between wild and captive François’ langurs. Conversely, the proportion of Bacteroidota was notably lower in the white-headed langurs than in the François’ langurs, whereas it was significantly higher in wild than in captive François’ langurs. However, the relative abundance of Mycoplasmatota was the third highest in captive François’ langurs and was markedly higher than that in wild François’ langurs.

**Figure 1:**
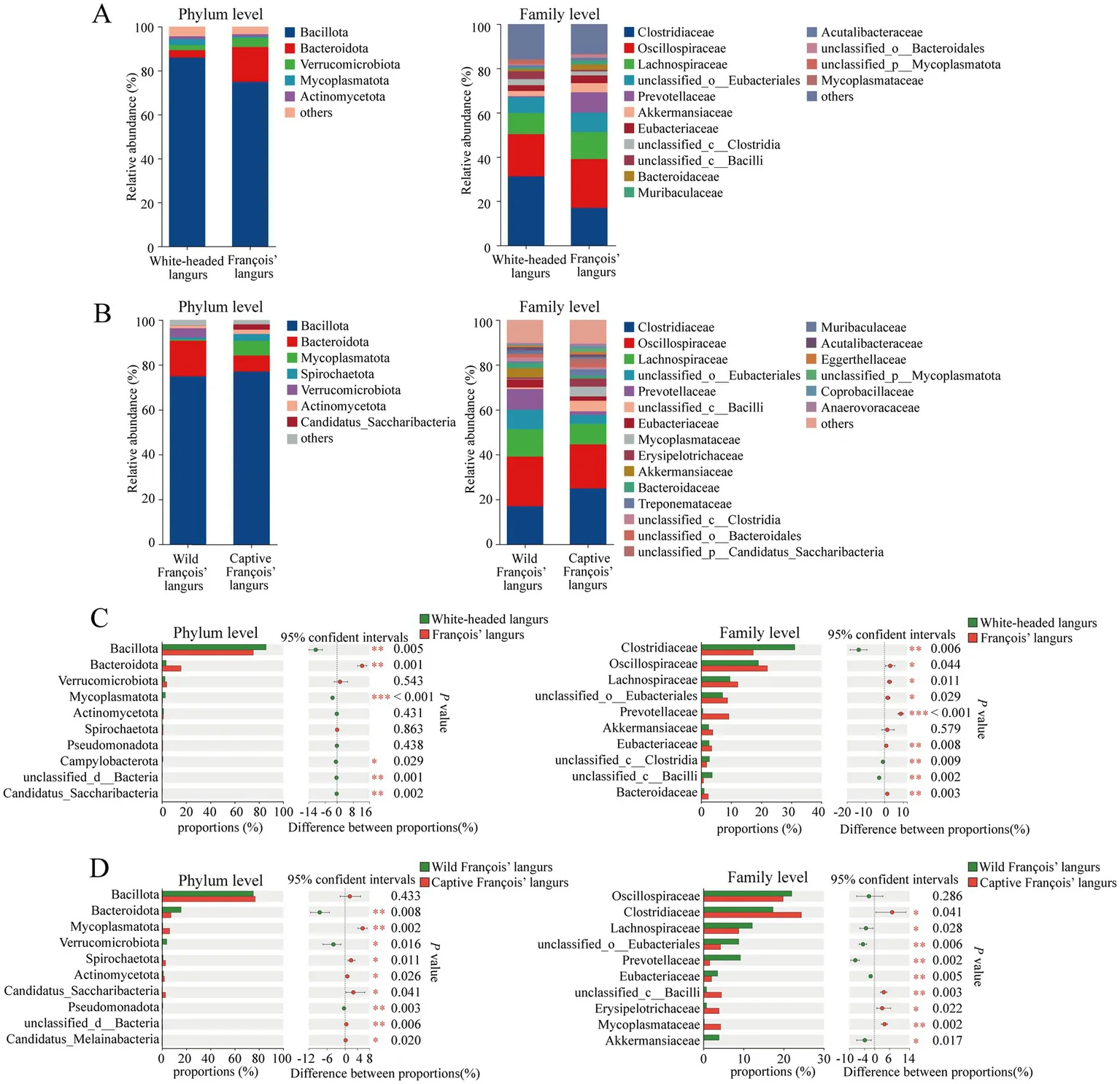
Differences in the gut microbiota based on metagenomic sequencing at phylum and family levels. Community composition of the gut microbiota in **(A)** white-headed langurs (number of samples, *n* = 9) and François’ langurs (*n* = 10), and **(B)** wild (*n* = 10) and captive François’ langurs (*n* = 8) at phylum and family levels are displayed. The “others” includes all taxa whose relative abundance is less than 1%. Significant differences in the composition of the gut microbiota between **(C)** white-headed langurs and François’ langurs, and **(D)** wild and captive François’ langurs at phylum and family levels, as determined by GLMMs, are shown. * *p* < 0.05, ** *p* < 0.01, *** *p* < 0.001.

At the family level, the gut microbiota was dominated by Clostridiaceae (31.11% ± 5.24%), Oscillospiraceae (19.03% ± 3.32%), and Lachnospiraceae (9.58% ± 1.53%) in white-headed langurs (Figure 1A). The dominant taxa of wild François’ langurs were Oscillospiraceae (22.01% ± 2.35%), Clostridiaceae (17.32% ± 5.19%), and Lachnospiraceae (12.17% ± 1.18%) (Figures 1A,B). The predominant taxa of captive François’ langurs were Clostridiaceae (24.39% ± 7.00%), Oscillospiraceae (19.84% ± 7.55%), and Lachnospiraceae (8.79% ± 4.06%) (Figure 1B). The relative abundance of Clostridiaceae was significantly higher in white-headed langurs, whereas the relative abundances of Prevotellaceae, and Bacteroidaceae were lower than those in François’ langurs (Figure 1C). In addition, the proportions of Prevotellaceae, and Akkermansiaceae were markedly higher in wild François’ langurs than in captive individuals (Figure 1D). Other gut microbial composition at the phylum and family levels are shown in Supplementary Tables S3, S4.

The alpha diversity of gut microbiota significantly differed between white-headed langurs and François’ langurs; however, no significant differences were observed between wild and captive François’ langurs (Figures 2A,B; Supplementary Tables S5, S6). For instance, the Simpson, Ace and Chao indices of white-headed langurs were significantly higher than those in François’ langurs, and the Shannon index was lower in white-headed langurs. The result from PCoA based on Bray-Curtis distance also revealed significant separation in the community structure of the gut microbiota between two groups (Figures 2C,D).

**Figure 2:**
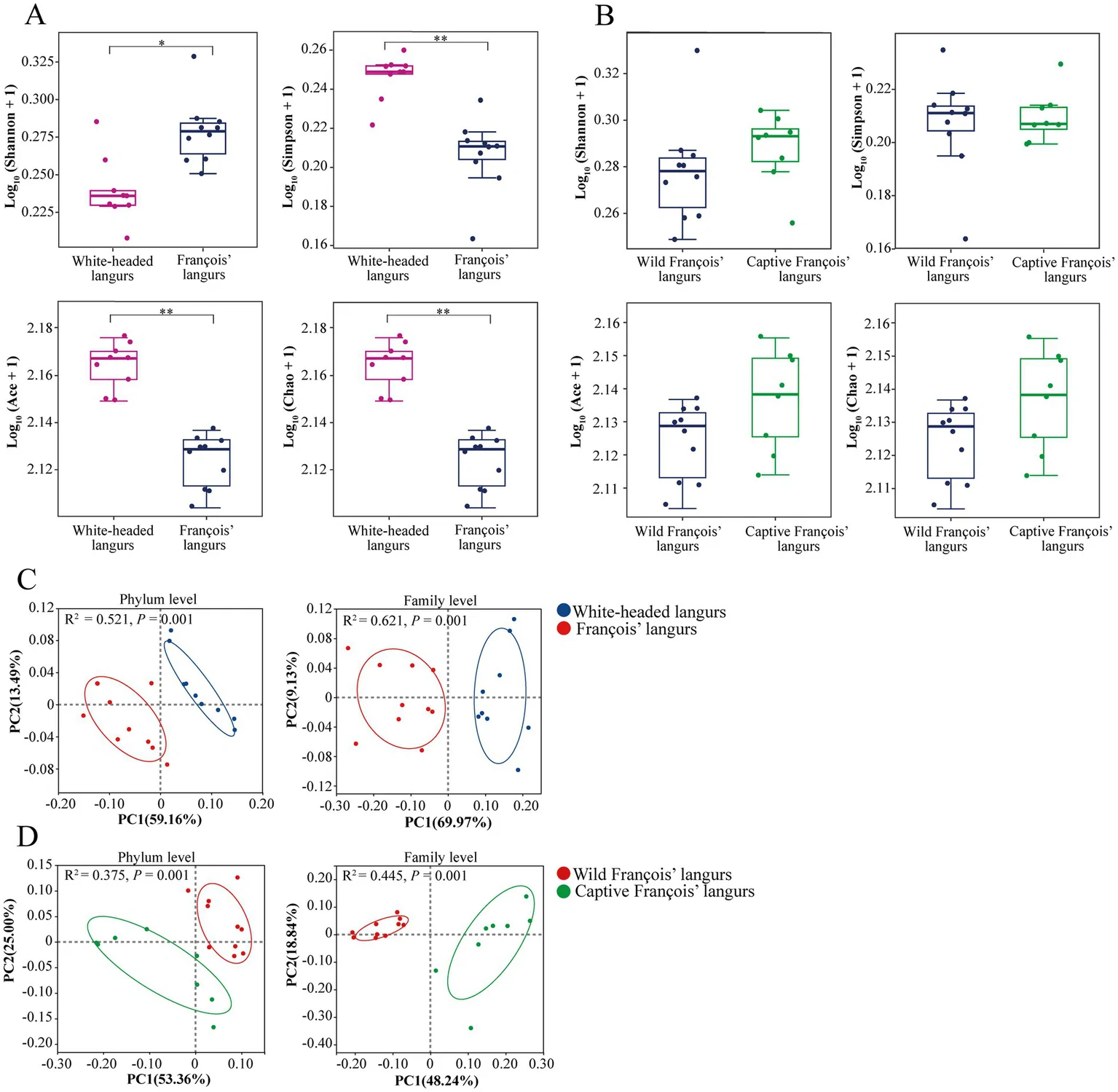
Differences in alpha diversity (Shannon, Simpson, Ace and Chao indices) in the gut microbiota of **(A)** white-headed langurs (*n* = 9) and François’ langurs (*n* = 10), and **(B)** wild François’ langurs (*n* = 10) and captive François’ langurs (*n* = 8). PCoA analysis based on Bray-Curtis dissimilarity in the community structures of **(C)** white-headed langurs and François’ langurs, **(D)** wild and captive François’ langurs at phylum and family levels are displayed. * *p* < 0.05, ** *p* < 0.01, *** *p* < 0.001.

### 3.3 Differences in functional profiles of gut microbiome

According to KEGG pathway annotation results, the functional genes of all samples were predominantly associated with Metabolism. The proportion of functional genes in gut microbiome associated with Metabolism at Level 1, Global and overview maps, Carbohydrate metabolism and Amino acid metabolism at Level 2, ko01100 (metabolic pathways), and ko01110 (biosynthesis of secondary metabolites) at Level 3, and M00035 were significant higher in wild François’ langurs than white-headed langurs and captive François’ langurs (Figure 3; Supplementary Tables S7, S8). At module level of KEGG pathway, the genes related to M00001, M00003, and M00165 were more enriched in white-headed langurs than François’ langurs (Figure 3A). However, M00001, M00003, and M00165 had higher proportion in captive than wild François’ langurs (Figure 3B). Other functional annotations of the gut microbiome are shown in Supplementary Tables S7, S8.

**Figure 3:**
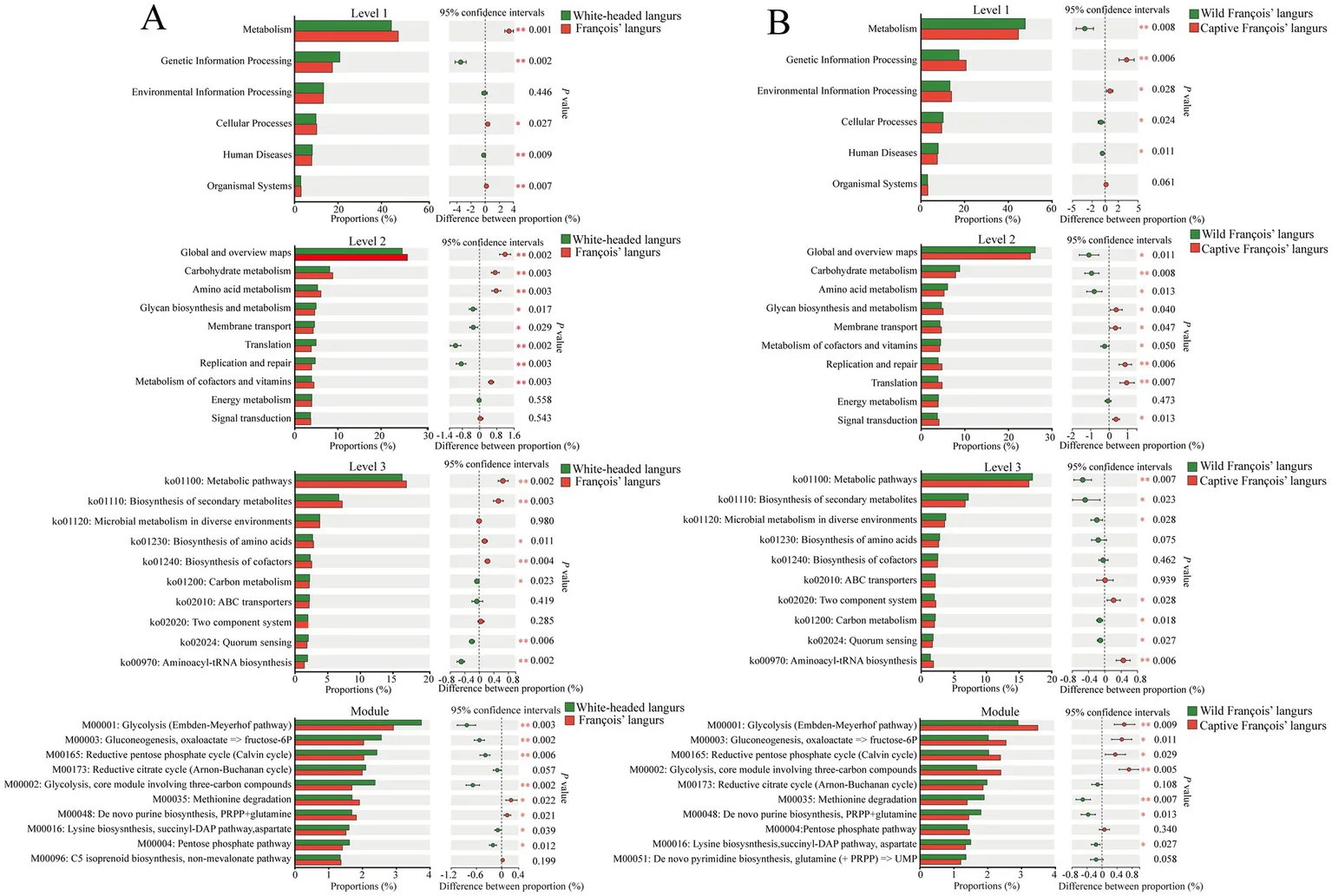
Differences (by GLMMs) in the relative abundance of functional genes at KEGG pathways in **(A)** white-headed langurs (*n* = 9) and François’ langurs (*n* = 10) and **(B)** wild François’ langurs (*n* = 10) and captive François’ langurs (*n* = 8) at Level 1, Level 2, Level 3, and Module. * *p* < 0.05, ** *p* < 0.01, *** *p* < 0.001.

The results of PCoA showed significant differences in KEGG pathways between white-headed langurs and François’ langurs (Level 1: R^2^ = 0.616, *p* = 0.001; Level 2: R^2^ = 0.650, *p* = 0.001; Level 3: R^2^ = 0.651, *p* = 0.001; Module: R^2^ = 0.647, *p* = 0.001) (Figure 4A). No significant differences in the gut microbiome were observed between captive and wild François’ langurs, except for the module level (Level 1: R^2^ = 0.065, *p* = 0.302; Level 2: R^2^ = 0.055, *p* = 0.361; Level 3: R^2^ = 0.126, *p* = 0.110; Module: R^2^ = 0.233, *p* = 0.006) (Figure 4B).

**Figure 4:**
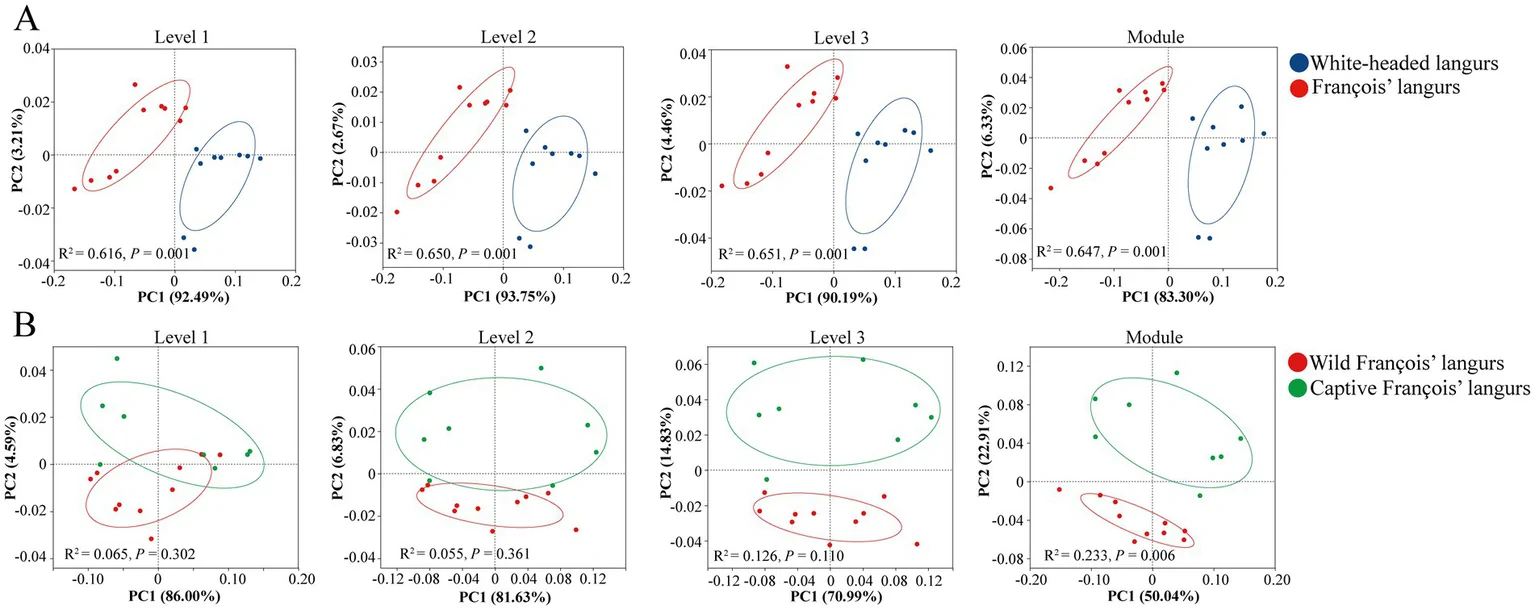
PCoA based on Bray-Curtis dissimilarity for **(A)** white-headed langurs (*n* = 9) and François’ langurs (*n* = 10), and **(B)** wild François’ langurs (*n* = 10) and captive François’ langurs (*n* = 8) at Level 1, Level 2, Level 3, and Module of KEGG pathways.

In the CAZymes database, Glycoside Hydrolases (GHs) accounted for the highest proportion. For GHs and the GH2 family, their proportions were significantly higher in François’ langurs than white-headed langurs, as well as in captive François’ langurs. However, the relative abundances of GTs and the GT28 were lower in François’ langurs than white-headed langurs and captive François’ langurs (Figures 5A,B; Supplementary Tables S9, S10).

**Figure 5:**
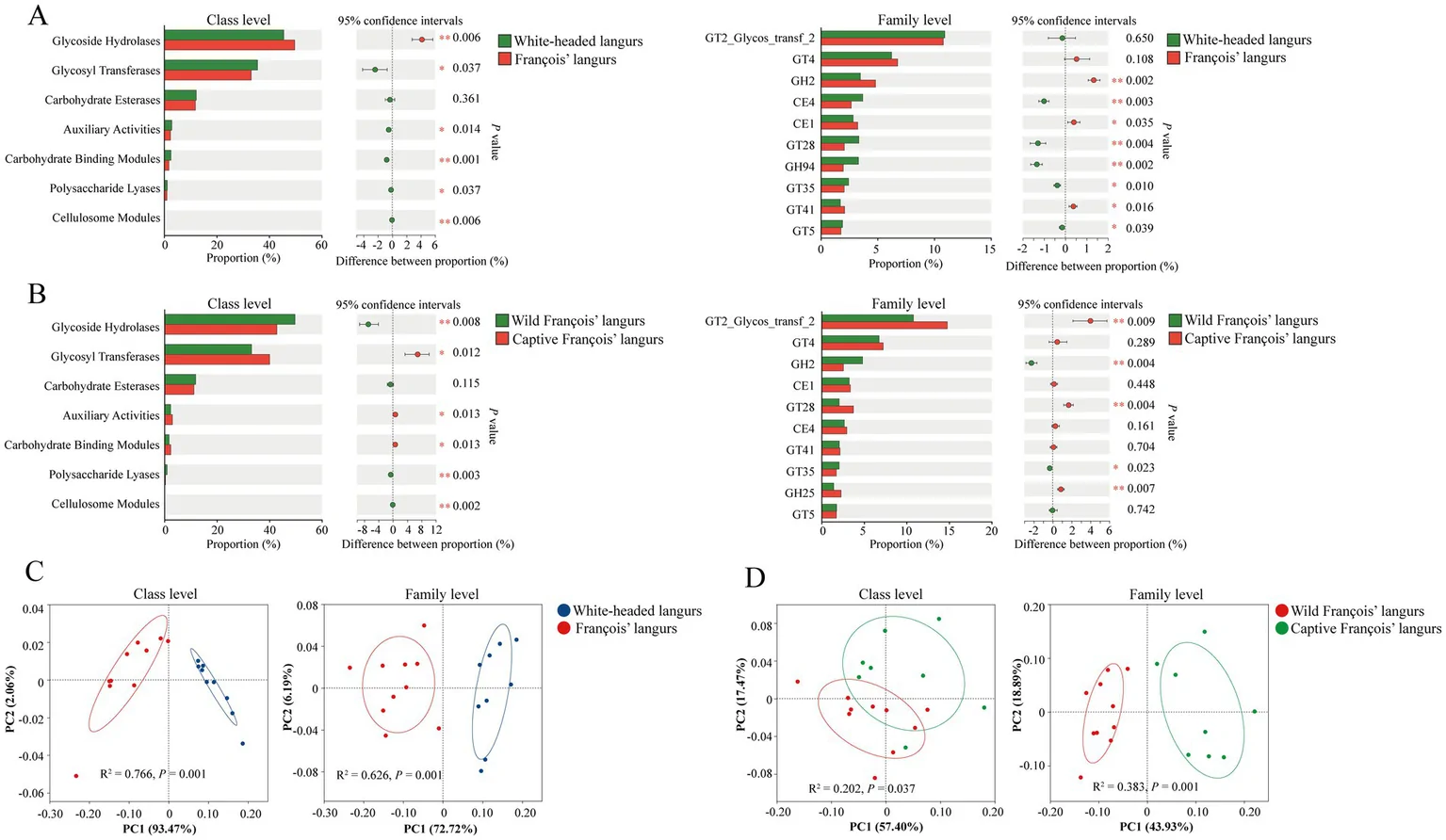
Differences (by GLMMs) in the proportion of gut microbial functional genes in CAZymes for **(A)** white-headed langurs (*n* = 9) and François’ langurs (*n* = 10), **(B)** wild François’ langurs (*n* = 10) and captive François’ langurs (*n* = 8) at class and family levels. PCoA based on Bray-Curtis dissimilarity in the gut microbiome of **(C)** white-headed langurs and François’ langurs, and **(D)** wild François’ langurs and captive François’ langurs at CAZymes class and family levels are showed. * *p* < 0.05, ** *p* < 0.01, *** *p* < 0.001.

The results of PCoA analysis indicated that significant differences were observed at class and family levels in all groups (white-headed langurs vs. François’ langurs: R^2^ = 0.766, *p* = 0.001 at class level; R^2^ = 0.626, *p* = 0.001 at family level; wild François’ langurs vs. captive François’ langurs: R^2^ = 0.202, *p* = 0.037 at class level; R^2^ = 0.383, *p* = 0.001 at family level) (Figures 5C,D).

The antibiotic resistance genes (ARGs) were dominated by antibiotic target alteration, antibiotic efflux and antibiotic target protection at the resistance mechanism level, Multidrug, glycopeptide, tetracycline and peptide at the Antibiotic class level, *macB*, and *efrA* at ARO name level. The relative abundances of ARGs including antibiotic efflux, multidrug and *optrA*, were higher in wild François’ langurs than white-headed langurs and captive François’ langurs (Figure 6; Supplementary Tables S11, S12). In addition, tetracycline-related genes (*tetA (58)*) were more enriched in captive François’ langurs than wild François’ langurs (Supplementary Table S12).

**Figure 6:**
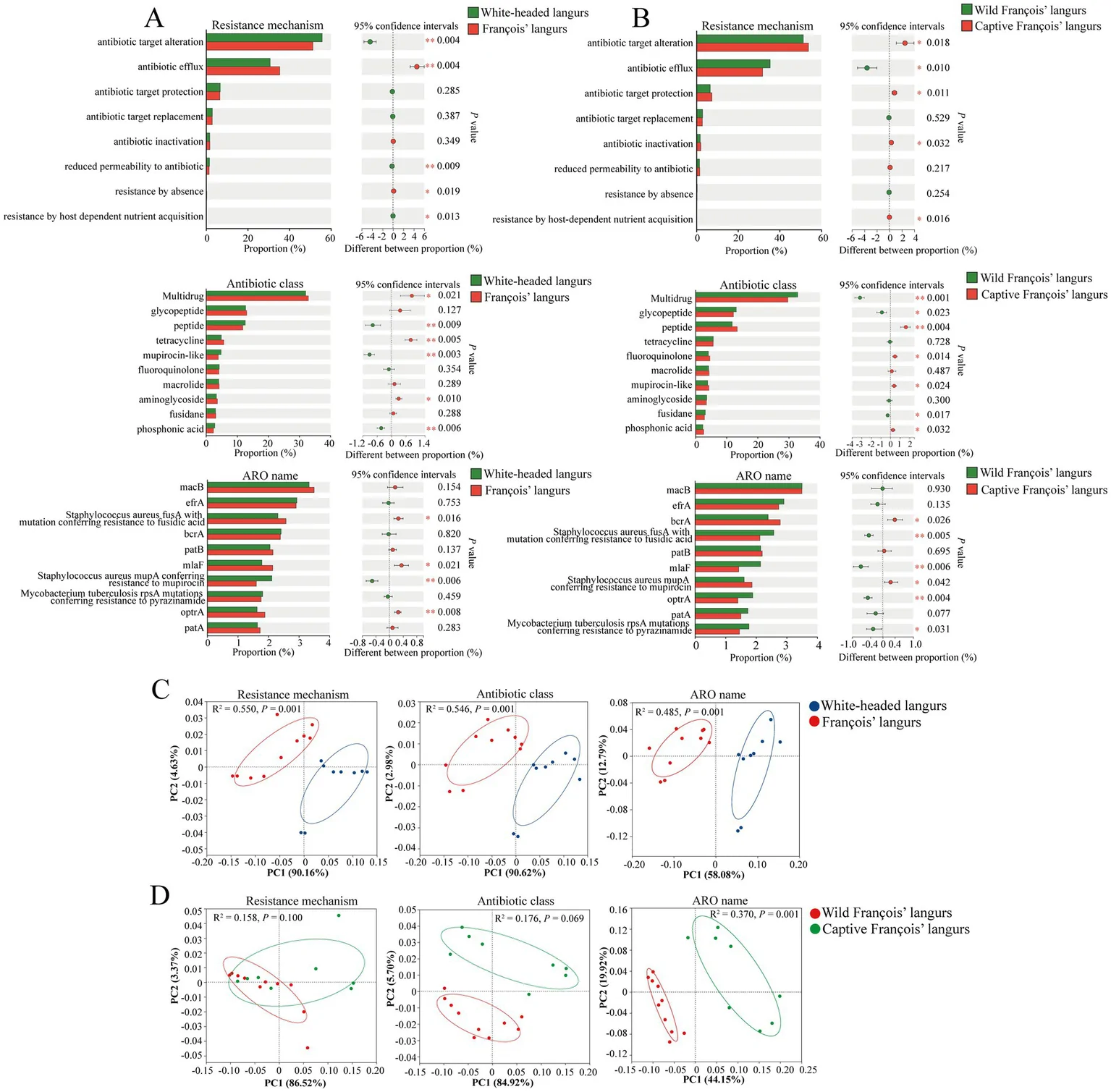
Differences (by GLMMs) in the relative abundance of functional genes in the gut microbiome based on CARD database for **(A)** white-headed langurs (*n* = 9) and François’ langurs (*n* = 10), **(B)** wild François’ langurs (*n* = 10) and captive François’ langurs (*n* = 8) at resistance mechanism, antibiotic class, ARO name level. * *p* < 0.05, ** *p* < 0.01, *** *p* < 0.001. PCoA based on Bray-Curtis dissimilarity in the gut microbiome of **(C)** white-headed langurs and François’ langurs, and **(D)** wild François’ langurs and captive François’ langurs at resistance mechanism, antibiotic class, ARO name level.

The PCoA results revealed significant differences in the ARGs profiles between white-headed langurs and François’ langurs at three levels, and significant difference at ARO name level between wild and captive François’ langurs (white-headed langurs vs. François’ langurs: R^2^ = 0.550, *p* = 0.001 at resistance mechanism; R^2^ = 0.546, *p* = 0.001 at antibiotic class; R^2^ = 0.485, *p* = 0.001 at ARO name; wild François’ langurs vs. captive François’ langurs: R^2^ = 0.158, *p* = 0.100 at resistance mechanism; R^2^ = 0.176, *p* = 0.069 at antibiotic class; R^2^ = 0.370, *p* = 0.001 at ARO name) (Figures 6C,D).

## 4 Discussion

In our study, the composition of gut microbiota was dominated by Bacillota, which accounted for more than 70% of total gut microbiota. Similar patterns have been observed in colobinae primates, such as Guizhou snub-nosed monkeys (Hale et al., 2019), golden snub-nosed monkeys (Su et al., 2016), and Udzungwa red colobus monkey (*Procolobus gordonorum*) (Barelli et al., 2015). In order to consume foods rich in cellulose, colobinae primates’ digestive system possesses typical structures, including an expanded rumen and sharp molars, which can effectively ferment and digest fiber-rich diets (Huang, 2002; Matsuda et al., 2022). The animals absorb nutrients from the complex cellulose and hemicellulose via the gut microbiome, because they lack the appropriate enzymes to digest these substrates (Li et al., 2015; Wang et al., 2019). Bacillota makes important contributions to the depolymerization of dietary fiber, breaking it down into oligosaccharides or monosaccharides, consequently producing acetic acids, butyric acids and lactic acids (Ndeh et al., 2017; Briggs et al., 2021; Sun et al., 2023b). It also produces SCFAs to provide energy for the host, which plays a crucial role in the host lipid metabolism and the degradation of complex plant carbohydrates (Flint et al., 2012; Blaak et al., 2020; Xiao et al., 2020). In addition, the functional gene profiles were consistent with the composition of gut microbiome. For example, functional genes related to Metabolism, Carbohydrate metabolism, Amino acid metabolism, ko01100, ko01110, M00001, and M00003 were present at relatively high abundances in their gut microbiomes. These functional genes are mainly associated with metabolism processes and energy production, and the glycolysis is a major pathway of energy metabolism (Rigoulet et al., 2020). Glycoside Hydrolases had the highest abundance in all CAZymes genes, and these enzymes could hydrolyze the glycosidic bonds of carbohydrate substrates. Similar results have been observed in other ruminants, such as yaks (*Bos grunniens*), Alpine musk deer (*M. chrysogaster*), mainland serow (*Capricornis sumatraensis*), and white-lipped deer (*Cervus albirostris*) (Gong et al., 2020; Zhang H. et al., 2025). Hence, the composition and functions of gut microbiome are related to dietary habits (Frankel et al., 2019; Lee et al., 2023). The white-headed langurs and François’ langurs are typical species of genus *Trachypithecus*, they are highly folivorous primate and heavily depend on leaves, especially young leaves (Li et al., 2016; Chen et al., 2024). Previous studies have indicated that Assamese macaques (*M. assamensis*) and Hainan gibbons (*Nomascus hainanus*) also consume high proportion of leaves, the dominant bacteria of their gut microbiota are Bacillota (Liu et al., 2024; Lan et al., 2025).

The habitats of wild white-headed langurs and François’ langurs in the Guangxi Chongzuo White-Headed Langur National Nature Reserve are covered by limestone forests and are severely fragmented (Guangxi Forestry Department, 1993; Li B. G. et al., 2024). Due to the seasonal variations in plant phenology, food resources decrease in the dry season, consequently resulting in seasonal fluctuations in the diet of langurs (Huang et al., 2010). During periods when preferred foods are limited, primates adjust foraging strategies to utilize fallback foods (Serckx et al., 2015; Souza-Alves et al., 2021). The wild white-headed langurs and the wild François’ langurs predominantly consume leaves, fruits and others food items (Chen et al., 2024; Lai et al., 2024). They consume more mature leaves with higher cellulose content in response to the limited resources of preferred young leaves and fruits (Huang et al., 2010; Lu et al., 2021). Our results revealed that both alpha and beta diversity of the gut microbiota significantly differed between wild white-headed langurs and François’ langurs. Overall, the white-headed langurs depend on leaves more heavily than the wild François’ langurs (82.6% vs. 69.9%), with lower fruit consumptions (10.4% vs. 15.3%) (Chen et al., 2024; Lai et al., 2024). Commonly, the dietary richness is strongly related to gut microbiome diversity (Huang et al., 2022). Given the relative high fiber content in leaves, particularly the mature leaves, the white-headed langurs could have more complex diets, likely resulting in higher gut microbiota diversity than that of François’ langurs (Lai et al., 2024). In fact, heritable diet preferences are considered to be one of the mechanisms by which host-associated factors such as genetics influence the gut microbiota (Goodrich et al., 2014). Furthermore, the high abundance bacterial community and functional genes also had significant difference between the white-headed and François’ langurs. The relative abundance of Bacillota (the overall abundance of Oscillospiraceae, Clostridiaceae, Lachnospiraceae, and Eubacteriaceae) was significantly higher in the wild white-headed langurs than François’ langurs. These differences could be linked to their variations in their diets, because Bacillota plays an essential role in digesting dietary fiber and complex carbohydrates to provide energy for the host (Flint et al., 2012; Blaak et al., 2020; Xiao et al., 2020). In addition, feeding habits could be an important factor determining digestive enzyme activity and the colonization of gut microbiota (Yang et al., 2025). Due to the variation in dietary composition between the wild white-headed langurs and François’ langurs, interactions among host digestive tract morphology, digestive enzymes, and metabolism likely influence the composition and function of the gut microbiome. However, more detailed information is required.

Functionally, the pathways of metabolism were also significantly higher in wild François’ langurs, including carbohydrate metabolism and amino acid metabolism in Level 2, as well as ko01100 and ko01110 at Level 3. These differences suggest that the wild François’ langurs had a stronger metabolic capacity than the white-headed langurs in current study. The proportion of Glycoside Hydrolases showed a similar pattern, and GHs plays a similar role to ko01100 and ko01110, which are linked to carbohydrate metabolism. The GH2 family contains numerous genes encoding oligosaccharide degrading enzymes, such as β-galactosidase, which break down complex polysaccharides drive energy production and facilitate the synthesis of SCFAs (Gong et al., 2020). This reveals a synergistic effect between KEGG pathways and CAZymes in utilizing cellulose-containing plants, which may be associated with the hosts’ high-fiber diets. The results of PCoA based on Bray-Curtis distances indicated that significant separation in the functions at all levels between wild white-headed langurs and François’ langurs. Additionally, previous studies have reported that the gut microbiome of both species differs in its seasonal variation (Chen et al., 2023; Liu et al., 2023). For instance, the gut microbiota (including Bacillota and Oscillospiraceae) of François’ langurs maintains relative stability during seasonal variations, the proportion of most bacterial taxa did not significantly differ between the dry and rainy season (Liu et al., 2023). However, there were significant seasonal variations in the diversity and composition of gut microbiota in the white-headed langurs (Chen et al., 2023). According to the results of our study and previous findings, we suggest the structures and functions of gut microbiome exhibited marked differences between white-headed langurs and François’ langurs, which could be closely related to the host-associated factors (Goodrich et al., 2014; Bubier et al., 2021). Previous studies suggest that host physiological traits and evolutionary history significantly influence gut microbiome structure, and that microbiome divergence may reflect host evolutionary processes (Amato et al., 2019; Wang et al., 2026). The host-associated factors may include host phylogeny, ecological and functional traits, and host genes. Moreover, the white-headed langurs and François’ langurs are highly folivorous, with similar dietary composition that include leaves, fruits and others items (Chen et al., 2024; Lai et al., 2024). In this study, we collect all samples in the winter, and both langur species heavily depend on mature leaves in response to the shortage of preferred foods, likely resulting in limited difference in the dietary composition (Huang et al., 2010; Lu et al., 2021). Thus, variations in the gut microbiome of these langurs likely should not be accounted for by the differences in their diets. More detailed dietary data are needed to confirm this interpretation.

The influences of host on the gut microbiome are supported by the differences in the gut microbiome of the white-headed langurs and François’ langurs. Although the diets and environments of wild and captive François’ langurs differ, the compositions and functions of gut microbiome are similar in this study. For instance, the proportion of Bacillota did not differ between wild and captive François’ langurs, and alpha diversity was similar between the groups. These findings indicate that animals retain their core microbiome (Nishida and Ochman, 2018), and suggest that host-associated factors strongly shape the gut microbial composition (Goodrich et al., 2014; Bubier et al., 2021; Yang et al., 2021). Moreover, the gut microbiomes were functionally similar between François’ langur groups, showing similarities in Level 1, Level 2, and Level 3 of KEGG pathways, resistance mechanism and antibiotic class level of CARD. However, specific pathways or CAZymes exhibited marked differences, revealing that the pathways corresponding to metabolism (e.g., carbohydrate metabolism) and GHs (GH2) were markedly higher in wild langurs, likely due to their high-fiber diets in the wild populations (Barelli et al., 2015). Similar patterns are observed in provisioned and wild rhesus macaques (Chen et al., 2019), captive and free-range sika deer (*C. nippon*) (Wu et al., 2021). Actually, environmental factors have been regarded as the important factor in shaping gut microbiome, even outweighing the host-associated factors (Rothschild et al., 2018; Lee et al., 2023). In this study, the gut microbiome was similar between wild and captive François’ langurs, despite of their completely different living environments, likely highlighting the importance of host on the gut microbiome over their diets.

The ARGs constitute a fundamental mechanism underlying bacterial resistance to antibiotics (Tunyong et al., 2021). These ARGs are widespread in the whole environment, commonly observed in gastrointestinal tracts of human and animals (Wu et al., 2023; Feng et al., 2024). The gut microbiota serves as a reservoir for ARGs, which could be transfered and diffused through contacts among humans, animals and the environment (Li G. et al., 2024). In our study, the multidrug resistance-related ARGs were the highest abundance in both groups, showing that François’ langurs had more enriched multidrug resistance genes (MDRs) than white-headed langurs and captive François’ langurs, and the wild François’ langurs may be regarded as potential reservoir of MDR genes. The proportions of ARGs (*mlaF, optrA*) were also markedly higher in wild François’ langurs than other langurs, which is potentially linked to the habitat of the wild François’ langurs (Pal et al., 2016; Li Y. et al., 2023). *MlaF* and *OptrA* confer resistance to linezolid and oxazolidinones, respectively, linezolid is a member of the oxazolidinones class of synthetic antibiotics (Cattaneo et al., 2016; Morroni et al., 2017; Kolich et al., 2020). The linezolid resistance genes are widely distributed in animals and humans, and *OptrA* has a high prevalence in the gut of poultry (Shen et al., 2024). Further, we observed that the habitats of wild François’ langurs were located nearby farmlands, and the local villagers utilized poultry manure in agricultures, which may make these langurs potentially exposed to antibiotics (Li Y. et al., 2023). This potential exposure risk could be closely associated with the higher proportions of ARGs observed in wild François’ langurs. The results of PCoA showed no significant difference between wild and captive François’ langurs at resistance mechanism and antibiotic class levels. Therefore, host-environment interactions influence the enrichment and difference of ARGs (Li G. et al., 2024). Further, investigation of the soil microbiome in the habitat may help clarify the links and exposure pathways between poultry manure and the ARGs of the wild François’ langurs.

In our study, we examined the influence of host-associated factors and the environment on the gut microbiome by analyzing the composition and function of the gut microbiome of two limestone forest-dwelling langur species using metagenomic sequencing. Although this study provides evidences of the influences of host -associated and environmental factors, several limitations should be acknowledged. For example, we collected samples of the wild langurs without individual identifications, likely affecting microbial diversity. However, previous study indicated that pooled sampling is an efficient and economical approach, and no significant difference in gut microbiota diversity is observed between pooled and individual samples (Cox et al., 2022). In addition, host-associated factors include genetics, physiology, digestive morphology and behavior. Hence, interspecific difference in gut microbiome is not exclusively attributed to host, making the host-associated and environmental factors cannot be completely separated. To distinguish the influence of host-associated factors from that of the environment, we controlled for potential variables as much as possible. Our findings show a stronger influence of host-associated factors than the environment in shaping the composition and function of the gut microbiome. As the research remains largely descriptive, future studies should strengthen physiological analyses to provide direct evidence on the relative importance of host and environmental factors in shaping the gut microbiome.

## 5 Conclusion

The study showed that host-associated factors have a stronger influence than environmental factors on the gut microbiome of langurs. The core gut microbiome maintains relative stability, and accounts for a large proportion of the microbial community across different species and environments. Significant differences were observed in alpha diversity and the proportion of Bacillota in white-headed langurs and François’ langurs. Interestingly, the significant differences of alpha diversity and the proportion Bacillota between wild and captive François’ langurs were absent. In wild François’ langurs, the functional genes of carbohydrate metabolism and Glycoside Hydrolases showed greater enrichment than other langurs, which could be related to their high-fiber diets. Further, the proportion of ARGs related to multidrug were more enriched in wild François’ langurs, which is likely related to the poultry manure and antibacterial biocides. These findings provide more evidences for host-associated and environmental factors impact on the gut microbiome of animals, which improves our understanding of potential driving factors underlying ARGs, offering a theoretical basis for the future conservation of langurs.

## Data Availability

The raw sequencing data present in this study can be found in online repositories. The name of the repository and accession number can be found at: National Center for Biotechnology Information (NCBI), BioProject ID: PRJNA1453454, https://www.ncbi.nlm.nih.gov/.
