# Supplementary material for: Gut microbiome profiles of two limestone forest-dwelling langurs show stronger influence of host-associated factors than environment
Source: Front Microbiol. 2026 Sep 30;17:1953526. doi: 10.3389/fmicb.2026.1953526 (PMC13661472; doi:10.3389/fmicb.2026.1953526)
Supplement: Supplementary file 1 [file Table_1.docx]

**Table S1 Information on all fecal samples in metagenomic sequencing**

| Sampling host | Living environment | Sampling time | Sampling site | Number of samples | Sample number |
| --- | --- | --- | --- | --- | --- |
| White-headed langurs (*Trachypithecus leucocephalus*) | wild | 2020.11.29 | Banli | 9 | ZWY01 - 09 |
| François’ langurs (*Trachypithecus francoisi*) | wild | 2020.11.20 | Duozhu | 10 | TH146 -155 |
| François’ langurs (*Trachypithecus francoisi*) | captive | 2020.12.25 | Wuzhou | 8 | WZ002. WZ003. WZ005. WZ006. WZ010. WZ012. WZ013. WZ015 |
